# Hurdles to the Adoption of Gene Therapy as a Curative Option for Transfusion-Dependent Thalassemia

**DOI:** 10.1093/stcltm/szac007

**Published:** 2022-03-10

**Authors:** Isabelle Thuret, Annalisa Ruggeri, Emanuele Angelucci, Christian Chabannon

**Affiliations:** Department of Pediatric Onco-Hematology, Center for Hemoglobinopathies, La Timone Hospital, Marseille University, Marseille, France; Hematology and Bone Marrow Transplant Unit, IRCCS San Raffaele Scientific Institute, Milan, Italy; Eurocord, Hopital Saint Louis, Paris, France; EBMT Cellular Therapy and Immunobiology Working Party, Leiden, the Netherlands; Hematology and Cellular Therapy, IRCCS Ospedale Policlinico San Martino, Genova, Italy; EBMT Cellular Therapy and Immunobiology Working Party, Leiden, the Netherlands; Institut Paoli-Calmettes Comprehensive Cancer Centre, Inserm CBT-1409, Aix-Marseille Université, Marseille, France

**Keywords:** thalassemia, hematopoietic cell transplantation, hematopoietic cellular therapy, gene therapy, gene editing, quality of life

## Abstract

Beta-thalassemia is one of the most common monogenic disorders. Standard treatment of the most severe forms, i.e., transfusion-dependent thalassemia (TDT) with long-term transfusion and iron chelation, represents a considerable medical, psychological, and economic burden. Allogeneic hematopoietic stem cell transplantation from an HLA-identical donor is a curative treatment with excellent results in children. Recently, several gene therapy approaches were evaluated in academia or industry-sponsored clinical trials as alternative curative options for children and young adults without an HLA-identical donor. Gene therapy by addition of a functional beta-globin gene using self-inactivating lentiviral vectors in autologous stem cells resulted in transfusion independence for a majority of TDT patients across different age groups and genotypes, with a current follow-up of multiple years. More recently, promising results were reported in TDT patients treated with autologous hematopoietic stem cells edited with the clustered regularly interspaced short palindromic repeats-Cas9 technology targeting erythroid BCL11A expression, a key regulator of the normal switch from fetal to adult globin production. Patients achieved high levels of fetal hemoglobin allowing for discontinuation of transfusions. Despite remarkable clinical efficacy, 2 major hurdles to gene therapy access for TDT patients materialized in 2021: (1) a risk of secondary hematological malignancies that is complex and multifactorial in origin and not limited to the risk of insertional mutagenesis, (2) the cost—even in high-income countries—is leading to the arrest of commercialization in Europe of the first gene therapy medicinal product indicated for TDT despite conditional approval by the European Medicines Agency.

## Available Options for Standard of Care TDT Treatment

Beta-thalassemia is an inherited hemoglobin (Hb) disorder resulting from at least 250 mutations in the beta-globin gene that lead to decreased (beta+ genotype) or absent (beta0 genotype) synthesis of the beta-globin chain. The resulting imbalance in alpha/beta chains produces anemia, mainly driven by ineffective erythropoiesis. Beta-thalassemia represents a group of disorders which constitute a considerable medical, psychological, and economic burden in a wide range of countries, including many with limited resources and an underdeveloped healthcare infrastructure.^1^

In transfusion-dependent thalassemia (TDT), the most severe form of beta-thalassemia, patients require lifelong transfusion and iron chelation therapy due to anemia present since infancy. With the proper administration of supportive care, the life expectancy of TDT patients has dramatically improved since the 1970s. Over the past 20 years, the introduction of orally active iron chelators and increasing use of cardiac magnetic resonance imaging in detecting presymptomatic cardiac iron overload have improved the prevention of both mortality due to heart failure (the leading cause of death in TDT patients before these advances) and morbidity.^1-3^ Additionally, an increased understanding of the molecular mechanisms underlying both dyserythropoiesis and iron overload resulted in the development of new drugs that alleviate disease phenotype.^4^ The current life expectancy of TDT patients is greater than 50 years with optimal monitoring and treatment of iron overload.^5,6^ Nonetheless, quality of life (QOL) is still greatly impaired by iron overload-related morbidity—particularly in older patients—residual anemia despite regular transfusions and required adherence to lifelong treatment.^7,8^ Furthermore, access to complex, multidisciplinary, and expensive care is only available in high-income countries, while a large number of patient candidates live in low- to middle-income countries.

Allogeneic hematopoietic stem cell transplantation (allo-HSCT) has been used to treat TDT for over 40 years, demonstrating its capacity to cure an inherited globin disorder.^9-12^ It remains the only viable curative treatment and is increasingly used outside of western countries; however, it is limited by a lack of related/unrelated donors for a large proportion of patient candidates. The European Society for Blood and Marrow Transplantation (EBMT) and other groups have reported excellent results in transplant patients younger than 14 years with an HLA-matched sibling donor (MSD).^12^ In most countries, MSD HSCT is a standard of care in children with TDT; it should be performed before iron overload occurs in order to decrease the risk of transplant-related mortality. Several studies have demonstrated that the quality of treatment that patients received throughout their life before allo-HSCT impacts the results of HSCT, by protecting tissues from chronic iron-related oxidative stress.^13^ Thalassemia-free survival (TFS) now routinely reaches >90%, especially when cord blood transplantation from an HLA-identical sibling donor is used resulting in a low incidence of acute and chronic graft versus host disease (GVHD).^14^ Excellent results were also reported in pediatric patients receiving transplants from 10/10 HLA-matched unrelated donors with 2-year overall survival and TFS similar to those obtained with MSD, despite a higher incidence of acute and chronic GVHD.^12,15^ In addition to HSCT with HLA-identical donors, the use of alternative donors was recently reported when using transplants from haploidentical transplantation yielded encouraging results,^16^ while an excess of graft failure was reported by international registries for unrelated cord blood transplantation.^17^ Allogeneic HSCT alleviates the need for lifetime supportive care and improved QOL,^18,19^ provided that the risks of transplant complications are lowered with transplantation at a young age and appropriate care in the pretransplant period.

Adult TDT patients remain a challenge for transplantation with a high mortality rate (>30%) in initial reports.^20,21^ A 2016 EBMT report analyzed over 1500 TDT patients with transplants after year 2000, of whom only 5% were adults. The mortality rate has improved in the adult cohort, but still approached 20%.^12^ This improvement was ascribed to better clinical conditions for adult patients compared to previous cohorts, resulting in an increased tolerance to conditioning regimen toxicity and allo-HSCT itself. It follows that adult TDT patients, who have received optimal transfusion and chelation therapy with lifelong control of iron toxicity, represent an appropriate population for gene therapy—a strategy that does not entail the risk of immunological complications associated with allo-HSCT.^22^

Patients living with TDT have impaired health-related quality of life (HRQL). Studies conducted over 1-2 years or longer, up to more than 20 years, after allogeneic transplantation have reported physical and emotional improvements in HRQL.^18,19,23,24^ Negative associations between older age or chronic GVHD and HRQL scores were reported.^19^ Furthermore, HRQL scores of patients living in high-income countries and who are cured of thalassemia after allo-HSCT have improved when compared with those of patients treated with blood transfusions and iron chelation therapy.^18,24^

## Gene Therapy for TDT: Transitioning from Clinical Trials to Clinical Adoption Reveals the Treatment Landscape’s Complexity in Genetic Diseases

Gene therapy recently emerged as an alternative curative option for children and young adults with thalassemia and without HLA-identical donors. It has the potential for phenotypic correction through either expression of a functional copy of the beta-globin gene in hematopoietic stem cells and their progeny or through enhanced expression of gamma chains that assemble with alpha chains to produce fetal hemoglobin.^25,26^Table 1 summarizes the results of completed and ongoing clinical trials that have evaluated these 2 approaches in TDT patients. These efforts spanning the past two decades have culminated in the first conditional approval by the European Medicines Agency (EMA, 2019) of a gene therapy medicinal product (GTMP) indicated for TDT (betibeglogene autotemcel or beti-cel). Gene therapy by addition of a functional beta-globin gene has been explored in a series of academia and industry-sponsored clinical trials. All these attempts relied on the use of self-inactivating lentiviral vectors (SIN-LV), which are now preferred over gammaretroviral vectors for safety reasons (reduced risk of insertional mutagenesis). The use of SIN-LV results in a semi-random integration of the transgene into the host genome. Among SIN-LV, the HPV569 vector and the next generation BB305 or LentiGlobin vector bear a “marker” in the nucleotide sequence coding for the gene therapy-derived hemoglobin (HbA^T87Q^) that differentiates it from endogenous or post-transfusion hemoglobin (additionally, HbA^T87Q^ has antisickling activity in Sickle Cell Disease, but this is beyond the scope of this review). The first TDT patient was treated with the HPV569 vector in 2007 as part of the LG001 trial.^27^ The patient suffered from beta+/beta0 TDT (HbE/beta0 thalassemia). Transfusion independence associated with benign clonal dominance was achieved. However, after 9 years, a decline in Hb levels has been reported and the patient required occasional red blood cell transfusions.^25^ Since this first report, results from phase I/II trials using the BB305 vector were published. A myeloablative regimen containing a PK-adjusted busulfan dose was administered before beti-cel injection in 22 TDT patients; transfusion independence was reached for the majority of non-beta0/beta0 patients.^28^ Next, improvements in transduction procedures with increases in both vector copy number and the proportion of transduced CD34+ cells in the phase III HBG-207 trial (NCT02906202) resulted in transfusion independence in 20/22 non beta0/beta0 pediatric and adult TDT patients, with no severe adverse events related to the experimental product reported.^29^ The results of these clinical trials were the basis for the conditional approval of the aforementioned first-in-class GTMP for non-beta0/beta0 TDT patients, aged 12 years or older and without HLA-identical sibling donors. Results were less satisfactory in beta0/beta0 TDT patients treated in the HGB-204 trial. However, the results of the HGB-212 trial (NCT03207009) that evaluated an improved manufacturing process based on the same transduction technology—similar to the HGB-207 trial—demonstrated transfusion independence in 12/14 beta0/beta0 TDT patients.^29^ Longer-term data suggest a stable response as transfusion independence was maintained in all patients who achieved it, sustaining HbA^T87Q^ levels for up to 7 years. This gives hope for a one-time curative treatment with a favorable long-term safety profile.^30^ Liver iron concentrations improved after successful gene therapy and several patients were able to stop chelation therapy. Indeed, any claim to cure the disease requires not only transfusion independence with no or mild anemia but also mitigation of dyserythropoiesis and restoration of iron homeostasis—as demonstrated with allo-HSCT, even when mixed chimerism is present.^31^ Patients treated with beti-cel for non-beta0/beta0 genotypes who reached transfusion independence also showed improvements in ineffective erythropoiesis markers such as plasma levels of soluble transferrin receptors, the hepcidin/ferritin ratio, and erythropoietin levels (in correlation with hemoglobin levels), and in myeloid/erythroid ratios assessed by bone marrow biopsy.^28,32^ These results need to be confirmed with a longer follow-up.

**Table 1.** Clinical trials that evaluate gene therapy medicinal products for TDT.

Study/ Clinical trial	Gene therapy medicinal product or lentiviral vector	Strategy	Genetic engineering technology	Conditioning regimen	Manufacturing conditions	Status	Sponsor	Results	Reference, Follow-up (Data cut off for analysis)
LG001	HPV569	β-Globin gene addition encoding ^T87Q^ β-globin	Self-inactivating lentiviral vector	MAC (Bu)	Academic Point of care manufacturing (POC)^b^	Completed	Bluebird bio	3 patients infused. 1 TDT patient stopped TF with stable Hb levels of 8.5-9 g/dL and benign clonal dominance that persisted 8 years, after which the patient requires occasional TF	(27, 25) follow-up 10 years (2017)
HGB-204 NCT01745120 phase 1/2	Lentiglobin/betibeglogene autotemcel/ BB305 vector	β-Globin gene addition encoding ^T87Q^ β-globin	Self-inactivating lentiviral vector	MAC (Bu)	Industry Central Manufacturing Organization (CMO)	Completed	Bluebird bio	18 patients treated. TF independence^a^ achieved for: 8/10 non beta0/beta0 patients 4/8 beta0/0 patients	(28, 30) median follow-up > 60 months (March 2021)
HGB-205 NCT02151556 phase 1/2					Academic POC^b^	Completed	Bluebird bio	4 patients treated. TF independence^a^ achieved for: 3/3 non beta0/beta0 patients- 0/1 beta0/beta0 patient	(28, 30) median follow-up > 60 months (March 2021)
HGB-207 NCT02906202 phase 3					CMO	Active not recruiting	Bluebird bio	23 non beta0/beta0 patients infused. TF independence^a^ achieved for 20/22 evaluable patients	(29, 30) median follow-up of 24 months (March 2021)
HGB-212 NCT03207009 phase 3					CMO	Active not recruiting	Bluebird bio	14 beta0/beta0 patients infused TF independence^a^ achieved for 12/14 evaluable patients	(29, 30) median follow-up of 23 months (March 2021)
NCT02453477 phase 1/2	GLOBE vector	β-Globin gene addition	Self-inactivating lentiviral vector	MAC Reduced toxicity (Treo/thiotepa)	Academic POC	Completed	IRCCS San Raffaele	9 patients treated. 4/9 (4/6 children) evaluable patients achieved TF independence	(33, 34) follow-up up to 60 months (April 2021)
NCT01639690 phase 1	TNS9.3.55 vector	β-Globin gene addition	Self-inactivating lentiviral vector	Non myelo-ablative (Bu at reduced doses)	Academic POC	Active not recruiting	MSKCC°	4 patients treated, all resumed TF	(35, 36) follow-up up to 7 years
THALES NCT03432364 phase 1/2	ST-400	Downregulation of erythroid BCL11A enhancer	Gene editing by Zinc Finger Nuclease	MAC (Bu)	CMO	Active not recruiting	Sangamo Therapeutics	3 patients infused. Minor increase in HbF after engraftment, all patients resumed TF	(41) follow-up of 2, 6, and 9 months (November 2019)
CLIMB THAL-111 NCT03655678 phase 1/2	CTX-001	Downregulation of erythroid BCL11A enhancer	Gene editing by CRISPR-cas9	MAC (Bu)	CMO	Recruiting	Vertex/CrispR Therapeutics	15 patients infused. Increased HbF (ranging from 4.5 to 13.5 g/dL) after engraftment allowing arrest of TF	(39, 40) mean follow-up 9.8 months (March 2021)

Abbreviations: MAC: Myeloablative Conditioning regimen, Bu: busulfan, Treo: treosulfan, TF: transfusion, CMO: Central Manufacturing Organization, POC: point-of-care manufacturing, TDT: transfusion-dependent thalassemia.

Tf independence was defined as weighted average Hb ≥ 9 g/dL without PRBC transfusions for ≥ 12 months.

For cell transduction step ° Memorial Sloan Kettering Cancer Center.

Transfusion independence was also reached in 4 of 6 pediatric patients (but none of the 3 adults) mainly bearing beta0/beta0 mutations, who were treated with the GLOBE vector in another trial conducted in Italy (NCT02453477). The investigators used a beta-globin gene addition strategy, with a conditioning regimen of thiotepa and treosulfan and intrabone injection of the graft.^33,34^ A smaller phase I clinical trial was conducted in the US (NCT01639690) and is mostly notable for being the only clinical trial that used a non-myeloablative-based conditioning regimen—all treated TDT patients failed to reach transfusion independence^35,36^ suggesting the need for sufficient myeloablation in preparation for engraftment of genetically modified autologous cells.

Gene engineering technologies that resort to transduction and transgene supplementation are likely to be challenged by gene editing technologies in the near future. Initial gene editing strategies either using clustered regularly interspaced short palindromic repeats (CRISPR)-Cas9 technologies or zinc finger nucleases target the expression of BCL11A, a key regulator of the normal switch from fetal to adult globin production that normally occurs during the first few months of life.^37^ The repair process with CRISPR-Cas9 involves the creation of insertions or deletions within the non-coding BCL11A erythroid lineage-specific enhancer on chromosome 2, thus downregulating BCL11A in erythroid precursors with no expected effect on other hematopoietic lineages.^38,39^ This non-coding change reactivates gamma-globin gene transcription and increases fetal hemoglobin protein concentrations in red blood cells. The aim is to replicate the natural expression of high concentrations of fetal hemoglobin in adulthood as in the hereditary persistence of fetal hemoglobin.

Early results have been reported from the industry-sponsored CLIMB-Thal-111 trial evaluating the safety and efficacy of CTX001, an investigational GTMP made from autologous CRISPR-Cas9-modified CD34+ hematopoietic stem and progenitor cells. Accrual in the CLIMB-111 is ongoing for TDT patients aged 12 to 35 years. Fifteen patients have been infused with CTX001 and stopped transfusions within 2 months after treatment. They remain transfusion-free as of last follow-up as a result of high levels of HbF production.^40^ The industry-sponsored THALES trial targets a similar objective while using a different gene editing technology. So far, 3 patients who did not achieve transfusion independence have been reported.^41^

A commonality across all clinical trials is that the starting material for medicinal product manufacturing is autologous peripheral blood (PB) stem cells obtained through apheresis of efficiently mobilized patients (HPC-A). The mobilization protocol associates G-CSF and plerixafor (used “off label” in this context) and allows for the collection of “megadoses” of PB CD34+ cells.^42^ With the exception of academic clinical trials, the starting material is shipped from collection/processing facilities at the treating hospital to a central manufacturing organization for further processing. The PB CD34+ cells undergo selection, are genetically modified ex vivo and then formulated and cryopreserved as a cell suspension, which qualifies as a GTMP. Upon release by the qualified pharmacist, using the results of in-process quality control and potency assays, the drug product is shipped from the manufacturing site back to the treating hospital for reception, temporary storage, thawing, and infusion. Thus, the use of a central manufacturing organization—as opposed to point of care manufacturing—often involves shipping the starting material from/the final product back to the treating hospital over long distances. As with every autologous living drug, consistency in the on-demand process does not guarantee product harmonization, nor does it exclude manufacturing failures or the possibility of out-of-specification products. Data regarding the actual manufacturing costs are not available, but information regarding the manufacturing of other types of gene therapies suggest that they are far below the price requested by the manufacturer and represent only a minor fraction of the price tag.

## Hurdles to the Adoption of Gene Therapy as a Curative Option for TDT

The results described above represent a remarkable achievement, and potentially offer new hope to patients affected with TDT as heralded by the conditional approval of the first-in-class GTMP. Nevertheless, recent developments in the field illustrate just how difficult it will be to provide broad access to such treatments.

Similarly to other GTMPs, such as autologous CAR-T cells, beti-cel has been approved based on data generated from small registration studies with limited follow-up and without being compared to a standard of care, whether supportive care or allo-HSCT. This mandates Post-Authorization Safety Studies and Post-Authorization Efficacy Studies in order to fully define safety and efficacy profiles, as illustrated by the recent description of several adverse events.

Two cases of secondary myelodysplastic syndrome (MDS)/acute myeloid leukemia (AML) were reported in two patients with sickle cell disease (SCD, another inherited globin disorder that could benefit from similar or comparable gene therapy approaches) included in the HGB-206 trial, which used the same BB305 vector than in the TDT HGB studies. An additional case of MDS was initially reported but later reconsidered as unrelated to the therapy. Further investigation led to the conclusion that the first case of MDS/AML, reported in 2020, likely resulted from busulfan exposure; monosomy 7 was present while no vector was detected in blast cells; monosomy 7 is frequently associated with myeloid malignancies induced by alkylating agents.^43^ Of note, a few cases of secondary MDS/AML derived from host cells associated with graft rejection have been reported after allo-HSCT for SCD.^44^ The second case was AML and led to the temporary suspension of accrual in the HGB-206 and HGB-210 SCD trials.^45,46^ The vector was present in leukemic cells that were not exposed to busulfan and insertional mutagenesis was suspected. The Marketing Authorization Holder soon thereafter communicated that the investigation revealed an insertion of the lentiviral vector sequence into the VAMP4 gene. VAMP4 has no known role in AML or cancer, and its regulation/expression was not affected. These observation do not support direct causality between vector integration and the development of leukemia.^47^ Indeed, the risk of insertional mutagenesis of SIN-LV is considered low compared to gammaretroviral vectors that have demonstrated considerable risk of leukemogenesis due to their preferential insertion near transcriptional start sites and proto-oncogenes.^48-50^ The presence of the erythroid-specific BB305 promoter probably decreases the risk of oncogene activation in other lineages.^51^ No cases of insertional mutagenesis leading to secondary leukemia were reported in clinical trials using SIN-LV until very recently (August 2021), when a patient developed secondary MDS after gene addition therapy for cerebral adrenoleukodystrophy (ALD) via the vector Lenti-D containing a MNDU3 promoter for ALD protein expression.^52^ The risk of leukemogenesis after gene therapy may be influenced not only by vector design but also by the host condition and the underlying disease. The baseline risk of hematological malignancies was reportedly higher in SCD patients than in the general population,^53^ possibly in relation to the chronic inflammation and stress erythropoiesis encountered in the disease.^46^ The hypothesis that a preexisting clonal hematopoiesis of indeterminate potential may progress to overt leukemia after gene therapy (or after unsuccessful allo-HSCT),^54,55^ raises the question of pretreatment screening in SCD patients. The use of hydroxyurea before and/or after gene therapy—while not associated with oncogenesis in the routine treatment of SCD patients—could be an additional risk factor. Until now, MDS/AML events or clonal dominance were not observed in thalassemia patients. Unlike SCD, thalassemia does not create a chronic inflammatory condition: no clear increased risk of MDS/AML has been identified, ineffective erythropoiesis encountered in TDT is partly suppressed by a regular transfusion regimen, and hydroxyurea is rarely used in TDT patients. At present, the benefit/risk ratio of treatment with beti-cel remains favorable for patients with TDT. This was also the conclusion of the EMA Pharmacovigilance Risk Assessment Committee evaluation performed after reporting of the 2 SCD cases.

Pricing and additional costs linked to supply chain complexity and patient care in the acute phase following treatment represent another hurdle to widespread adoption of these innovative therapies.^56-58^ For example, the direct cost of non-curative medical care for Italian TDT patients was calculated to be the equivalent of 31,833 USD/year in 2017.^59^ Worldwide, HSCT costs are highly variable depending on the country’s healthcare organization and complication rate. HSCT is nevertheless considered cost effective when compared to standard medical therapy since the cost amounts to nearly the same as the cumulated costs of standard medical therapy over 3 to 5 years. Gene therapy has also been proposed to be cost effective when compared to standard medical care,^60^ but it is argued that cost-comparison analyses of the curative options should be performed and subsequently a better comparator would be allo-HSCT. Only one paper compared US costs (not price) of HSCT and gene therapy (transduction step with academic point of care manufacturing)^61^ and concluded that gene therapy was 3 times more costly than HSCT. Following conditional marketing approval in Europe, bluebird bio—the manufacturer of Zynteglo (beti-cel)—asked for a price tag of 1 650 000 EUR or 1 900 000 USD while considering the savings in supportive care that 50 years of hypothetical thalassemia-free survival would represent.^62^

Beti-cel thus ranks among the most expensive therapies ever marketed. While bluebird bio offered to install annual payments over 5 years, and offered the condition of payment only upon demonstrated sustained clinical activity,^63^ the price of the medicinal product still appears prohibitive to public and private payers in many high-income countries, and is entirely unaffordable in low- to middle-income countries. Following conditional marketing authorization, payment or reimbursement conditions have not been approved in a single European country, preventing patients to receive this treatment. The German health system’s offer of a single payment of 950 000 USD with the condition of persistent clinical efficacy^64^ was refused by the company and—together with the lack of rapid decisions from several other major EU countries—triggered the discontinuation of its operations in Europe and the reorganization of its operations in the USA, as announced in a press release in August 2021.^65^ It is anticipated that bluebird bio will withdraw its marketing authorization for beti-cel from both the EU and the UK by early 2022.^66^ The U.S. Food and Drug Administration has accepted bluebird bio’s Biologics License Application for beti-cel for priority review.

A global framework for pricing negotiations for this new category of medicinal product is urgently needed. Claims from pharmaceutical companies of a treatment with lifelong efficacy will need to be further substantiated through the collection of reliable data in real-world conditions over extended periods of time. Global, transparent and open discussions must be conducted concerning what is and is not acceptable in relation to drug pricing, development costs, manufacturing, and savings associated with sparing prolonged or lifelong supportive care in order to overcome reimbursement issues and allow innovative treatments to reach the market and enter clinical practice. Hopefully, competition between companies, developing technologies, and large-scale manufacturing will improve the situation, however, this objective appears out of reach in the short term.

Finally, as TDT is a chronic disease, long-term comparisons are warranted for gene therapies with existing options such as allo-HSCT (Table 2). Such evaluations should focus not only on survival and morbidities but also on resource consumption and HRQL based on self-reported patient outcomes (health economic studies).^6,61^ Numerous health authorities recommend using cost-utility analyses for the economic evaluation of new treatments based on not just the quantity but also the QOL gained from healthcare interventions. An improvement in QOL after gene therapy is expected given the successful achievement of transfusion independence and lack of GVHD. Preliminary short-term results in children and adolescents who achieved transfusion independence in the HGB-207 and 212 studies demonstrated clinically meaningful improvement in child-reported PedsQL 4.0 total scores, especially in those with low baseline values.^67^ Until long-term data become fully available, the selection of patients who will benefit from receiving gene therapy will represent a significant challenge. To this end, the Italian Society for Thalassemia and Hemoglobinopathy (SITE) recently published an algorithm for patient selection and prioritization.^22^ In conclusion, while gene therapies appear to represent a major therapeutic advancement in the treatment of thalassemia, they raise the critical issues of long-term efficacy and safety, accessibility, and affordability. All patients treated with gene therapy in clinical trials must be rigorously followed in long-term studies, in order to allow for the evaluation of long-term disease control, the risks of secondary malignancies, and how the technology used in genetic engineering affects these risks.

**Table 2.** Comparison of mechanisms underlying the clinical activity, available toolboxes, and medical and societal constraints associated with HSCT versus gene addition versus gene editing in TDT.

	HSCT	GA	GE
Intervention-mechanism of action	Replacement of the entire hematopoietic system including immune system	“Random” integration of a functional beta-gene into each genome. Planned impact on only the erythropoietic system	Single targeted edit at desired location. Planned impact on only the erythropoietic system
Product/type of construct	Allogeneic hematopoietic cells	Gene insertion by a lentiviral vector	Gene editing by non-viral ex vivo CRISPR-Cas9 creating an insertion or deletion in specific regions
Available experience	Thousands in real-world setting	Approximately <100 as participants in clinical trials	Approximately 30 as participants in clinical trials
Donor required	Yes	No	No
Risk of acute and chronic GVHD (consequences of alloreactivity)	Yes	No	No
Stem cell processing	Minimal (non-substantial) cell processing	Substantial cell processing, including genetic engineering	Substantial cell processing, including genetic engineering
Myeloablation required	Yes	Yes	Yes
Immunosuppression required	Yes	No	No
Risk of insertional mutagenesis or oncogenic events	No	To be determined	To be determined for oncogenic events
Risk assessment	Well defined	To be defined	To be defined
Long-term follow-up	>40 years	Up to 7 years	Up to 2 years
Cost issue	No Cost equivalent to 3-5 years of medical standard treatment.	Yes Cost equivalent to 50 years of medical standard treatment^a^	Yes

Abbreviation: HSCT: hematopoietic stem cell transplantation; GA: gene addition; GE: gene editing, GVHD: graft versus host disease.

For betibeglogene autotemcel.

## Data Availability

No new data were generated or analyzed in support of this research.
